# Systemic Factors Fuel Food Insecurity Among Collegiate Student-Athletes: Qualitative Findings from the Running on Empty Study

**DOI:** 10.3390/nu17142254

**Published:** 2025-07-08

**Authors:** Barbara Gordon, Natalie Christensen, Jenifer Reader

**Affiliations:** 1 Nutrition & Dietetics, College of Health, Idaho State University-Meridian, Meridian, ID 83642, USA; barbaragordon@isu.edu; 2 Athletic Department, Idaho State University-Pocatello, Pocatello, ID 83209, USA; nataliechristens1@isu.edu; 3 Nutrition & Dietetics, College of Health, Idaho State University-Pocatello, Pocatello, ID 83209, USA

**Keywords:** food security, food insecurity, student-athlete, college athlete, college student, university student, student affairs, nutrition, contributing factors

## Abstract

Collegiate student-athletes are particularly vulnerable to food insecurity (FI). Prevalence rates range from 9.9% to 65%, although research is limited among this population. Background/Objectives: The challenge of balancing academic and degree progression requirements with training and competition demands can increase the risk for FI among student-athletes. Furthermore, insufficient funds for food has been reported for student-athletes living both on campus and off campus. Methods: This qualitative study employed a phenomenological design and constructivist theoretical framework to explore the experiences of athletic trainers, sports dietitians/nutritionists, and other professionals working with student-athletes in identifying and addressing FI among student-athletes via a series of online focus groups. Results: Participants (*n* = 27, 12 public colleges) had ≥7 years of collegiate athletics work experience, and most had been in their current position for <3 years. Five approaches to FI screening emerged; specifically, no screening, screening varies by team/sport, informal screening, dietitian screening, and formal screening. Emerging social determinants of FI included financial challenges, competing priorities, cultural/societal impacts, limited life skills, and the food environment. All these factors precipitated on a systems level, including individual, team/athletic department, and university/societal tiers. Conclusions: Athletic department and university policies and budgetary decisions emerged as potential antagonists of food security among student-athletes. FI mitigation strategies for student-athletes must go beyond simply addressing individual factors. Obtainment of food security among collegiate student-athletes requires system changes at the team/athletic department and university tiers.

## 1. Introduction

The United States Department of Agriculture Economic Research Service (USDA ERS) stratifies food security status into four tiers [1]. High food security is defined as no food access concerns, and marginal food security is defined as one or two indicators or risk of food insecurity (FI), such as anxiety related to insufficient access. Low food security and very low food security are the two categories of FI. Low food security is defined as less-than-optimal quality, variety, and/or desirability of food options, and very low is defined as multiple disruptions in food intake and eating patterns [1]. Collegiate student-athletes are particularly vulnerable to FI [2,3,4,5], or inadequate access to enough nutritionally adequate food, acquired in socially acceptable ways, to support an active, healthy life [6]. Extant research examined FI rates among student-athletes based on their university’s National Collegiate Athletic Association (NCAA) Division. Division I includes the most competitive colleges [7]. These schools can provide athletic scholarships, which may cover tuition or housing and meal plans; they also typically offer other benefits, such as fueling stations that provide access to healthy snacks. Division I student-athletes may also be “walk-on” (non-scholarship) players [7].

A scoping review (18 studies; peer-reviewed articles, abstracts, and gray literature) reported a FI prevalence rate ranging from 9.9% to 65% among student-athletes in all NCAA Divisions [8]. Other studies surveyed only Division I student-athletes about FI [2,3,4,5,9,10]. In the fall of 2016, Poll et al. reported a 19% FI prevalence rate among males (*n* = 111, 56% football and 18% baseball) at a Southeastern Conference university [4]. A survey of rural southwestern schools (*n* = 78 respondents, primarily Caucasian females) found that 32% of student-athletes were food insecure during the 2018 academic year [2]. In the fall of 2019, Goldrick-Rab et al. reported that 24% of Division I student-athletes were food insecure in the past 30 days [3]. Over half of the students scored at the very low threshold for FI; they were reducing their food intake, skipping meals, and foregoing food for a day or more due to financial constraints [3]. In 2022, a cross-sectional study (*n* = 45, primarily Caucasian females) found that FI was higher among student-athletes at a northwestern university compared with the school’s general population (60% vs. 42%, respectively) [9].

Studies have demonstrated lower academic performance and grade point averages (GPAs) among food insecure college students [2,11,12,13,14,15]. GPAs have also been found to be significantly predictive of FI severity; athletes with higher GPAs were found to have a lower risk of severe FI (OR = 0.77; 95% CI 0.63, 0.93) [13]. For Division I athletes, slipping academic performance can potentially put athletic scholarships at risk [12]. Inadequate consumption patterns have been associated with student mental health issues, including depression and stress [16,17]. Poll et al. found a significant association between disordered eating and being a food-insecure, male student-athlete [4]. Specifically, FI was associated with food preoccupation (r_t_ = 0.336, *p* < 0.001) and keeping/hiding food in a gym locker (r = 0.272, *p* < 0.003). Wurster et al. found evidence supporting FI as a contributor to systemic inflammatory stress in the body, resulting in mental health issues and eating disorders (statistical significance not reported) [18].

FI can also impact physical health [12,13,14,15,16,17,18,19]. In a study at a rural Oregon university, students in fair and poor health (*n* = 5438) were more likely to be food insecure (OR: 2.08, 95% CI: 1.07–4.63) [19]. In student-athletes, inadequate dietary intake can negatively impact cardiac function as well as muscle contraction, growth, and repair; this adverse reaction, in turn, negatively impacts athletic performance [20]. Insufficient energy intake can also impact training and competition performance goals required for student-athletes [20]. Even if energy needs are met, student-athletes who consume inadequate intake of specific nutrients risk nutritional deficiencies that can cause suboptimal performance and increase the risk of injury and illness. Inadequate intake of calcium and vitamin D, for example, can increase the risk of bone stress injury [20]. Food insecure student-athletes may also experience Relative Energy Deficiency in Sport (RED-S), which is impaired physical and psychological functioning caused by inadequate dietary intake [21,22].

### 1.1. Unique Considerations for FI Risk Among Student-Athletes

The challenge of balancing academic and degree progression requirements with training and competition demands can increase the risk for FI among student-athletes. Academic mandates translate to busy schedules and interfere with mealtimes. Brown et al. reported that practice times for nearly half (45%) of student-athletes conflicted with dining room hours, preventing them from fully utilizing their meal plans [23].

Insufficient finances have also been found to contribute to FI risk. Just over half of student-athletes receive athletic scholarships (~57%) [24]. Larger sports teams, such as football and track, have more walk-on (non-scholarship) players. These players need paid work to cover living expenses, such as groceries. Of note, Reader et al. found a higher prevalence of FI among football and track athletes [5]. Christensen et al. found that 17% of female student-athletes worked [25]. Insufficient funds to purchase food has been reported as a problem for student-athletes living both on-campus and off-campus [3,23]. Brown et al. found that 18% of student-athletes reported that their university meal plan funds did not sufficiently cover the quantity of food needed for a day or across a full semester [23]. These students turn to inexpensive, non-nutritious foods that do not offer the nutrients required for optimal competitive performance [26].

Low nutrition literacy has also been identified as a risk factor for FI among college students. Low levels of nutrition literacy and food preparation skills have been identified as risk factors for FI at both public and private institutions [27,28]. Students at the University of Alabama who had lower food preparation skills and lower confidence in their cooking skills were found to have higher levels of very low food security [29]. Morgan et al. observed an increase in food secure behaviors among food insecure students who participated in a cooking class at Appalachian State University, although rates of food security did not change significantly [30]. Rates of both FI and stress improved among students enrolled in a nutrition and culinary education program [31]. Moore et al. observed a weak relationship between nutrition literacy and food security at Texas Woman’s University; however, they note study participants were enrolled in dietetics and other health professions programs, so their baseline level of nutrition literacy was likely higher [32].

Race/ethnicity may also be a contributing factor. A large surveillance system (*n* = 13,720 students at 27 Minnesota state colleges) reported a higher FI prevalence among Black/African American and Native Hawaiian/Pacific Islander students (43% and 36%, respectively) [33]. Reeder et al. reported similar results [34]. Interviews with a strength coach at a New England football team revealed that 21% of football players experienced an 8% loss in body weight during the season; the coach suspected that the cause was due to patterns of inadequate consumption [35].

The coronavirus (COVID-19) pandemic was also identified as a contributing factor to FI among student-athletes. Davitt et al. found that college students experienced increased degrees of FI related to COVID-19, even if they were living off-campus with parents [36]. Reader et al. suggested that the high FI prevalence rates in their study may be associated with surveying student-athletes post-COVID-19 [5]. In addition, COVID impacted the budgets of university athletic departments. Pandemic-related cancellations of revenue-generating sports events led to considerable budget shortfalls. Anecdotal knowledge reveals that these budgetary downfalls led to reduced scholarship funds, meal stipends, fueling stations, and sports nutrition programming for student-athletes. To accommodate athletes whose years of play were impacted by COVID-19, the NCAA allowed athletes on Division I teams to play for additional competition seasons and carry additional members on scholarship [37]. Schools, thus, allocated scholarship funds across a greater number of student-athletes, likely decreasing the scholarship amounts received by each athlete.

### 1.2. Study Aim

This study investigated the following research question: What factors contribute to a risk for FI among collegiate student-athletes during the academic year? Insights were gleaned from the experiences of athletic trainers, sports dietitians, and other professionals working with student-athletes in the Big Sky Conference about factors contributing to FI among student-athletes. A series of focus groups investigated FI screening protocols employed with student-athletes; athletic professionals’ depth of understanding of the interwoven relationship between FI and mental and physical health, athletic performance, and academic performance, and factors contributing to the risk of FI among this population.

## 2. Materials and Methods

A phenomenological design and constructivist theoretical framework were employed to conduct and analyze the findings of focus groups examining the experiences of sports dietitians, athletic trainers, and other health professionals working with student-athletes in the Big Sky Conference.

### 2.1. Participants

A purposeful sample (*N* = 106) of athletic professionals from the 14 Big Sky Conference Division I schools was invited to participate. An optimal participation sample size (*n*) of 25–30 professionals was determined using the “information power” concept, which purports that sufficient power of qualitative studies relates to the study aim, sample specificity, theoretical framework, discussion quality, and analytical strategy [38].

Participants were recruited via personal email communications and received up to three invitations. The recruitment email included a description of the study. Interested participants received a follow-up email with a secure link to an online consent form to complete before the focus group. After the focus group, participants received a monetary incentive (gift card for a nominal amount).

### 2.2. Materials and Procedures

A deductive content analysis approach was employed to guide the focus group discussions. A moderator’s guide for facilitating semi-structured discussions among participants was developed. This guide included 14 sequenced questions with associated probes. The first question focused on the definitions of food security and FI. The USDA ERS definitions of food security and FI [1] were displayed on a slide for review by the participants. The moderator read the definitions and asked participants to raise their hands if they were familiar with the definitions. Participants were also encouraged to ask any questions regarding the different degrees of food security/insecurity. Additional questions probed on the potential factors contributing to the phenomenon of FI among collegiate student-athletes. Content validity testing of the guide included review and critique by experts on the appropriateness of its content for athletic professionals and study objectives. Modifications were made based on this feedback.

Based on the research objectives, a pre-established list of codes was collaboratively developed for performing descriptive coding using a hybrid thematic analysis approach; new codes were added (deductive coding) during this step. A study codebook was generated to ensure coding rigor and consistency.

### 2.3. Data Collection

Six online, 90-min focus groups were conducted from July to December 2023 using the Zoom web conference tool; meeting entry was password-protected. Of note, the efficacy of Zoom as a platform for qualitative data collection has been confirmed [39]. The moderators shared their backgrounds and interests in the study at the beginning of the focus group.

Additional data collection tasks were conducted to learn how the student-athlete stipends are calculated and develop the school profiles. To gather information on the process of determining student-athlete stipends, in-depth interviews were conducted with financial professionals working in a university Athletic Department (informed consent was obtained from these interviewees). In addition, school location categories were based on the US Health Resources and Services Administration definitions of rural and urban population centers, school size on the Carnegie Classification of School Size, and school budgets were retrieved from school websites [40,41,42,43,44,45,46,47,48,49,50,51,52,53].

### 2.4. Qualitative Data Analysis

Given the narrow aim of the study coupled with the highly specialized insights of the participants, adequate power was achieved via in-depth exploration and analysis of the topic. This was achievable with a small sample size and, thus, qualitative data saturation was obtained because there appeared to be no additional insights beyond those collected [54].

Zoom videos were transcribed and cleaned (no identifiable data) and sent to the participants for member checking to ensure the accuracy of the transcripts [55]. Transcripts were loaded into Lumivero (2023) NVivo (Version 14) for coding and analysis. Content analysis included reviewing the data, categorizing codes into themes, reviewing themes, defining and naming themes, and producing a report with the findings. Codes and themes were reorganized during the inductive coding process, allowing for more precise terminology and better elucidation of the cohesive themes and other findings. Investigator triangulation was employed—two authors coded independently to enhance the credibility and validity of the results, and the third conducted a thorough review of coding decisions. Disagreements were discussed by all three researchers until a consensus was reached.

Descriptive statistics (number and percent) were calculated using Excel^®^ v. 2506, build 16.0.18925.20050 (Microsoft, Seattle, WA USA). Consolidated criteria for REporting Qualitative research (COREQ) were employed as a best practice reporting tool for reporting qualitative data findings; see Supplementary Table S1 [56].

### 2.5. Ethical Considerations

The research team was composed of three female registered dietitian nutritionists. One was practicing as a sports dietitian at an NCAA Division I college; the other two were faculty members in the Department of Nutrition and Dietetics at the same Big Sky Conference school. The collegiate sports community is a close-knit, protective group. Thus, the sports dietitian (N.C.), an insider of this closed group, was integral in recruiting participants. To limit bias, however, N.C. did not facilitate or participate in the focus groups. B.G. and J.R. facilitated the focus groups; B.G. was formally trained as a focus group moderator. The Idaho State University Institutional Review Board reviewed and approved the study protocol.

## 3. Results

Thirty participants were recruited; 27 attended the focus groups. They hailed from the 12 primary colleges in the Big Sky Division I Conference. All participating schools were public colleges or universities; most (8/12, 66%) were in urban settings with student populations > 15,000 (7/12, 58%). Six schools (50%) had Athletic Department 2022 budgets of $10 to $19 million; five (42%), $20 to $29 million. More than half (14/27, 52%) of the cohort self-identified as athletic trainers, 22% (6/27) as sports dietitians, 15% (4/27) as performance coaches, including strength and conditioning coaches, and 11% (3/27) as administrative athletic staff. Participants were primarily females (17/27, 63%). Nearly two-thirds (16/27, 59%) of the participants had never been collegiate student-athletes. Table 1 provides a profile of the schools and a demographic overview of study participants.

**Figure 1:**
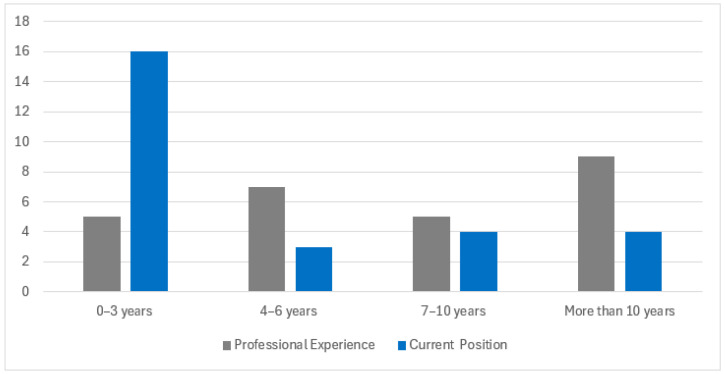
Comparison of the participants’ years of overall professional experience vs. the number of years in the current position.

One emerging finding was that as athletic professionals gained experience, they transitioned positions frequently. Most participants had ≥7 years of work experience in collegiate athletic department positions; however, most had been in their current positions for <3 years. Figure 1 illustrates that as the number of years of experience in the profession increased, the years of employment at the same school decreased.

### 3.1. Understanding of FI Among Professionals Working with Student-Athletes 

**Figure 2:**
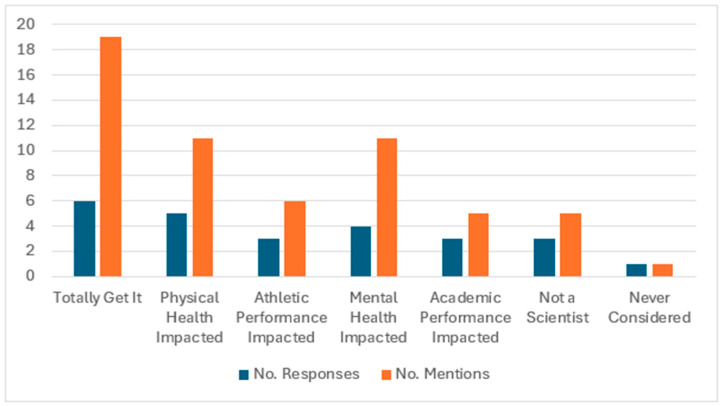
Participants’ self-reported level of understanding regarding the impact of FI on physical and mental health, and athletic and academic performance stratified by the number of responses.

All participants indicated that they were aware of the definitions of food security and FI. In response to a question about their level of understanding about the impact of FI on physical and mental health and athletic and academic performance, about one-quarter (6/27, 22%) of participants responded, “*totally get it*.” Some, however, explained that though they understood that there were connections, they could not explain the underlying physiological mechanisms connecting FI with health, academics, and athletic performance: “*I personally totally get it. It all makes sense to me, but I couldn’t get up there and teach on it. In the athletic training world, you see every side of it. You see the health side, obviously, some of the academic side, and you obviously see the mental health side. I think when you see enough of it and all those different situations, you can start putting 2 and 2 together*” (participant 026). Figure 2 provides the self-reported level of understanding for the study participants vis-à-vis the impact of FI on physical and mental health and athletic and academic performance stratified by the number of responses.

Though most of the participants had some understanding of the potential risk of FI on student-athlete health and performance, nominal time (mean = 7%, range = 1–30%) was spent discussing FI with student-athletes. Differences emerged based on participant positions; specifically, dietitians spent considerably more time than other professionals discussing nutrition and FI issues. Participant 010 noted, “*…that is not something we handle. We have a system to refer to the dietitian. It’s great*”.

### 3.2. FI Screening Protocols Employed with Student-Athletes

Five approaches to FI screening emerged; specifically, no screening, screening varies by team/sport, informal screening, dietitian screening, and formal screening. More than one-quarter of the participants (7/27, 25.9%) said they were unaware of any screening efforts at their school, coded as no screening. One assistant athletic director commented (003), “*I think we’re a little behind…We haven’t been looking at that*.” Eleven percent (11%, 3/27) noted that the decision to screen for FI varied by the college team and/or sport at their facility. Participant 007 illustrated this by explaining, “*But it’s really team by team. Or what’s the budget for each team? What can they afford to do*?” Nearly half of the schools (48.1%, 13/27) shared that student-athletes were “*informally screened*” regarding food availability/accessibility issues. A director of sports medicine (002), for example, said, “*We don’t have a formal way to evaluate food security status. I do think that’s an area of growth. I’m glad this conversation is happening because it gets the wheels turning in my head*.”

**Figure 3:**
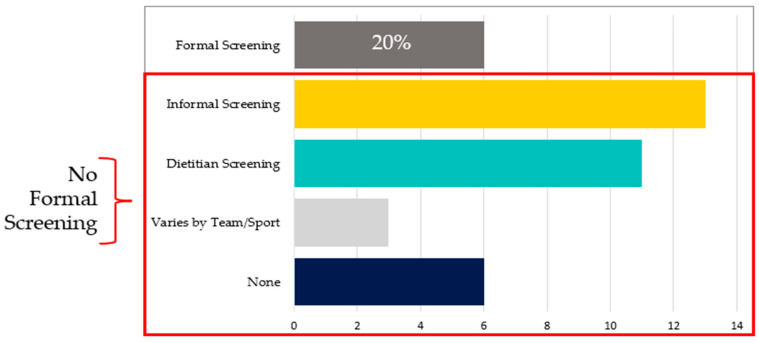
Breakdown of the five approaches to food security screening stratified by the number of participants reporting their school’s screening approaches.

Formal screening protocols varied. Many participants noted that potential food security concerns were referred to the team dietitian; of note, 40.7% (11/27) of responses indicated that screening fell into the scope of practice for the dietitian vs. their profession. One athletic trainer noted, “*The only other thing that I would add from an athletic trainer’s perspective is that if anyone mentions anything about food, like in the athletic training room, we just immediately refer to the dietitian. That’s really all we do*” (011). Reinforcing role differentiation, one sports dietitian shared, “*…as a dietitian, I have a lot of those conversations, and as you start to ask questions, you ask when are you eating and what are you eating? Those questions definitely come up*” (005). The inclusion of food security screening during the Preparticipation Physical Evaluation (PPE) was a practice shared by representatives of three schools. One noted, “*I helped out with physicals for students last spring, and we did do a food security screening during those exams*” (020). Some schools shared that they screen via survey tools, “*we created a validated survey that includes different nutrition knowledge questions. Part of that survey asks about food security*” (021). Another said, “*Every three years, we have our NCHA survey, and we’ve incorporated food security questions into that tool. Findings of the last survey found food insecurity rates similar to what is seen nationally*.” (020). Participant 029 emphasized the need for screening “*more often because life situations change*”. To determine total formal screenings conducted, the number of screenings by dietitians (11), sport team (3), PPE (3) school survey tool (1), and NCHA survey (1) where combined: 19/27 or 70.4% of participants reported some mode of formal FI screening among their student-athletes. Figure 3 provides a breakdown of the five approaches to food security screening stratified by the number of schools employing each screening approach.

### 3.3. Factors Influencing the Prevalence of FI Among This Population

Systems-level social determinants of FI for collegiate student-athletes fell into three tiers: individual, team/athletic department, and university/societal. Most of the comments focused on individual-related contributing factors.

#### 3.3.1. Theme 1: Individual Contributing Factors

Four subthemes precipitated for individual contributing factors: financial challenges, limited life skills, competing priorities, and cultural considerations (food preferences/familiarity). Nearly all participants (26/27, 96.3%) identified financial challenges as the primary factor for FI and limited finances as a key contributing factor. Participant 014 explained that financial struggles were the reason some student-athletes needed paying jobs, “*They have less resources, so after practice, they have to go to work to earn their support of their living costs*.” (014) Participants explained that needing to work was often related to resource distribution inequities, e.g., not all student-athletes receive scholarships/stipends. In contrast, participants mentioned the potential benefits of the 2024 NCAA Name, Image, Likeness (NIL) policy, “*It’s a huge, big game changer for athletes who can put their name on social media and say that they got a sponsorship. They can get a meal plan for 12 weeks and just get the food. But this is not the kind of athlete that already struggles. It is uneven like that*” (005). Other contributing financial factors included the cost of living off campus (6/27, 22.2%); one participant explained that scholarship/stipend funds are based on the cost of eating at the dining hall and not off-campus food costs. Participant 024 stated, “*The cost of living in our town has gotten ridiculous. I don’t think their stipend checks are enough to cover what they actually need.*” The final issue was food affordability among international students who may purchase more expensive imported foods from their home countries.

Limited life skills emerged as an individual contributing factor for FI risk. One participant shared, *“…at the end of the day, we’re working with young people who do not have a lot of life skills. I think that’s the biggest challenge*” (026). Precipitating themes included limited knowledge on how to eat healthy, grocery shop, prepare meals, manage money, and effectively manage time. More than half of the participants (15/27, 55.6%) identified challenges in making healthy choices as a contributing factor to FI risk, sharing insights such as, “*But I would also put up there that some of these kids don’t know how to make their own healthy choices of food… I just don’t know that they’re always going there and making the right choices*” (013). A comparable number of participants (15/27, 55.6%) highlighted limited grocery shopping and meal preparation skills as additional contributing factors. Lack of money management skills was another individual factor identified by participants (12, 44.4%). Finally, ten participants (10/27, 37.0%) felt that rather than the challenge of balancing competing priorities, student-athletes possessed less-than-optimal time management skills.

Competing priorities, or the challenge of balancing academics, athletics, work, and other life commitments, was another theme identified by participants as a contributing factor to FI risk (18/27, 66.7%). Participant 29 noted, “*I would say competing priorities… They do have the money to get food. But can they afford the most nutritious, nutrient-dense items?*” Participants described other examples of the busy schedules and disparate demands for typical student-athletes that impact meals. “*The cafeteria is only open so late, so if practice goes late… they’re trying to figure out something to eat.*” (025) Another emerging theme identified was that given their schedules, student-athletes often just don’t have the time to eat; about one-quarter of the participants (7/27, 25.9%) explained that for many student-athletes, eating is a low priority. One participant said, “*I have quite a few nursing program kids who really have a hard time juggling their schedules and cannot eat before practice…So, they’re skipping a meal or skipping a snack in between and they’re not getting the kcaloric intake that they should*” (006). Convenience was also a competing priorities theme; student-athletes opted for low-density, packaged, or fast-food options over nutrient-dense foods.

Finally, emerging cultural considerations included stigma, socioeconomic factors, and personal identity. Comments regarding stigma revealed positive and negative sentiments; schools were proactively combating stigma versus promulgating a culture in which student-athletes are reluctant to ask for help. The socioeconomics and personal identity themes highlighted student-athletes selecting familiar foods instead of nutrient-dense options, 8/27 (29.6%) and 7/27 (25.9%), respectively. Table 2 provides an overview of the individual factors contributing to FI among student-athletes as perceived by collegiate athletic professionals.

#### 3.3.2. Theme 2: Team/Athletic Department Contributing Factors

Three subthemes emerged regarding contributing factors specific to teams/athletic departments; these included finances, competing priorities, and culture. Finances included budgetary impacts, insufficient stipend funds, and resource distribution inequities, 10/27 (37.0%), 9/27 (33.3%), and 8/27 (29.6%), respectively. Participants shared that competing budgetary priorities translated to reduced funding for food, “*I think finances are the biggest problem… as budget cuts come around, food is the first thing to be cut from a team’s program. Whether it is the type of food or snacks they’re purchasing on the road, availability and type of food they get in the weight room, food is, unfortunately, the first thing to go as finances are limited*” (027). Insufficient stipends also emerged as a team/Athletic Departments, “*It also ties into living off campus, because if they live off campus, then they don’t eat at the dining hall and they get a very small stipend on Mondays to spend on food every week*” (016). Resources available varied by the college team; thus, the ability to fund FI initiatives varied for each team. One participant noted, “*The food that is given to the teams depends on the team’s budget. For instance, football coaches think nutrition is very important and they do a lot of fundraising around that through a foundation. And this allows the football team to have two meals a day, and it’s brought in by a catering group*” (019).

For teams/Athletic Departments, competing priorities emerged as “*eating is a low priority*” (10/27, 37.0%), inadequate funding for fueling stations (8/27, 22.2%), and sports nutrition staff (5/27, 18.5%). The “*eating is a low priority*” theme precipitated via the acceptance of scheduling conflicts between team practice times and cafeteria dining hours, “…*scheduling conflicts between practice time and the cafeteria is a huge one*” *(09).* Other examples were a lack of willingness among athletic departments to pay for fueling station stock and/or hire a sports dietitian/nutritionist due to competing budget priorities.

Team/Athletic Department culture also emerged as a contributing factor. Overall, cultural factors were mentioned 11 times (11/27, 40.7%); dialogue focused on Athletic-Department-wide sentiments of prioritizing food and nutrition. Both positive and negative sentiments emerged, as well as statements about the need for support from the administration to ensure food security among all student-athletes. Positive themes (6/27, 22.2%) reflected Athletic Departments taking proactive actions to increase access to food. Participant 008 noted, “*…our student-athletes need more accessibility to food and we’re trying to push that... We’ve started stocking areas, especially high traffic areas like our weight room…we are looking for a fueling station…it has been a huge push for us… We’ve identified an area that our student-athletes need, and we’re trying to move in the right direction*”. Similar to financial factors, negative themes (5/27, 18.5%) related to department leadership not prioritizing three things: supplemental food for student-athletes, fueling stations, and sports nutrition staff. Participant 026 shared, “*I’d say with nutrition, they didn’t see the investment and the payoff for buying more food…*” The general sentiment among administrators (4 directors and 3 assistant directors), who responded “*I totally get it*” to their understanding of the relationship between FI and mental and physical health, athletic performance, and academic performance, was frustration. One said, “*I’m on the administrative side, so I look at the money we’re spending, and there are so many priorities across the department. It’s hard to think about putting a lot of money into something that’s going to help half the student population. If we knew exactly what that half of the student population needed, we’d be more than happy to invest in a way that could really help them. I think a key to this is trying to get students to feel comfortable asking for help, the specific help they need, and then helping them in the way they need. We try to put these big overarching educational programs in place, and it might hit 10 or 15% of them, and it might not. We chase our tails a lot trying to figure out how to resolve a problem when we don’t know exactly what each student’s issue is*” (023). Table 3 provides examples of team/Athletic Department-related factors contributing to FI among student-athletes as perceived by collegiate athletic professionals.

#### 3.3.3. Theme 3: University/Societal Contributing Factors

On the university/societal tier, emerging subthemes included financial challenges, competing priorities, and cultural/societal factors. Financial challenges involved the lack of quality options in dining halls and/or food pantries. More than four-fifths of the participants (23/27, 85.2%) shared that student-athletes were concerned that dining room options did not provide the nutrients needed for optimal performance, sharing comments such as, “*every athlete will tell you that our cafeteria is not good*” (001). As noted above, dissatisfaction with food pantry options also emerged as a contributing factor. Given that stipend budgets are part of the university-wide budgeting process, insufficiency of these monthly funds also emerged as a contributing factor under this theme. One participant said, “*And then, it’s not that they don’t know the type of foods they need to be eating, it’s that they’re trying to stretch the limited funds they have left. So, they’re making choices like fast food, because they can get more meals out at the same amount of money, even though they know it’s not the best thing for their performance or their overall health*” (024). Cost of living emerged as a financial factor for students living off campus; participants raised concerns about the high housing costs and the insufficiency of the stipends.

Competing priorities emerged as a theme for the university/societal tier in that the food environment reflected that “eating is low priority.” Food availability concerns (7/27, 25.9%) related to on-campus dining options not being open during hours matching the time frames when the studentathletes have time to eat. Food access/proximity (4/27, 14.8%) themes surfaced regarding international students; specifically, access to familiar foods and the need for transportation to travel to stores with culturally relevant food options. Given that university services include the provision of food plans, dissatisfaction with the food environment could be a reflection that “*eating is a low priority*” vis-a-vis university funding priorities. The food environment, thus, falls into both the financial challenges (quality of options) and the competing priorities (availability, access/proximity) themes.

Precipitating cultural/societal factors included local socioeconomic considerations, stigma, school culture, and social media/advertisements. Participant 026 discussed the need for universities to take into consideration the local economy, “*…trying to work with the university to put a very realistic number on… what’s that gonna cost you in the community where you are…*” Stigma (4/27, 14.8%) also emerged as a theme, as it can prevent student-athletes from applying for food assistance benefits, utilizing the campus food pantry, or obtaining enough food during team road trips, especially since the perception among university cohorts that student-athletes have sufficient funds and access to food. Participant 026 elucidated the issue by saying, “*We have an on-campus food pantry…But honestly, we don’t advertise it a lot…we’ve actually gotten complaints from campus that some people are overusing it…*” During travel, given that food is provided, stigma precipitates via student-athletes feeling judged for being hungry between meals/snacks. Participant 005 said,” *We’re doing some things to reduce the stigma around food insecurity, and it has been really helpful.*” School culture (3/37, 11.1%) emerged regarding the recognition that mitigating FI is a university-wide problem requiring the engagement of leaders and administrators beyond Athletics. Participant 008 noted, “*I definitely think that the university has identified that a lot of our student-athletes need more accessibility to food… We’ve started stocking areas, especially in our high traffic areas, like our weight room… [but] a team that’s not in here frequently, they still may not get the full amount of food that they’re looking to get.*” Social media and advertisements (2/27, 7.4%) were mentioned regarding student-athletes “*trying or attempting various fad diets…. [seen on] TikTok or Instagram… And it ties into funds. They don’t have the funding… to execute those different types of diets to the point where they’re going to be successful at this level of an athlete*” (002).

Table 4 provides examples of the university/societal factors contributing to FI among student-athletes as perceived by collegiate athletic professionals. Other cultural/societal factors were the influence of stigma and social media on dietary intake and food choices.

**Figure 4:**
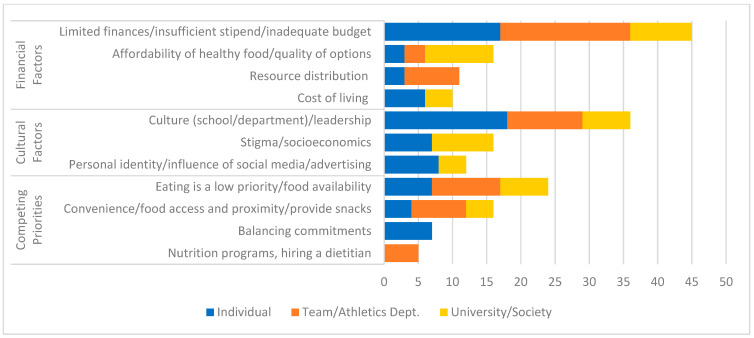
Breakdown of major and minor contributing factor themes stratified by percentage of mentions during focus groups.

Predominant overlapping themes for individuals, team/Athletic Department, and university/societal tiers were financial challenges, competing priorities, and cultural/societal considerations. Financial factors were by far the most frequently discussed by the participants. Competing priorities also emerged as a theme for the three groups—each group prioritized other items above the food environment. The two most prominent themes fell into these two tiers: budget (insufficient resources, stipend, funds for quality food), and eating is a low priority for athletes, Athletic Departments, and schools. Cultural/societal factors also spanned the three groups; the need to address values emerged for all three groups. Figure 4 provides a breakdown of the categories of contributing factors stratified by the percentage of mentions during the focus groups.

### 3.4. Calculation of Student-Athlete Stipends

Determination of student-athlete stipends varies by university and state regulations.

The financial professionals interviewed explained that stipends are derived from the average cost of living for the general student population. In their state, the school board was involved in establishing stipend guidelines. Based on those requirements, the university housing office determines the average (tier 2) meal plan and the average cost of campus living; combined, those two amounts equal the stipend amount provided for student-athletes living off-campus. Individual teams can add supplemental funds to the stipend pool; however, those monies come from individual team budgets.

Based on this state’s stipend requirements, for fiscal year 2025, one school’s stipend was $4211 per semester for fiscal year 2025. This equates to about $1060–1100/month for both food and lodging. In that school’s location, the average gross rent is $861/month, which leaves student-athletes with $199–239/month for food. Given the 6% sales tax on food in that state, these student-athletes only have $187–225/month for food. The typical food cost per person in this city, however, was $368/month. The stipend, thus, covers 51–61% of the average monthly food cost.

## 4. Discussion

This study employed a phenomenological design and constructivist theoretical framework to explore the experiences of athletic trainers, sports dietitians, and other professionals working with student-athletes to identify the social determinants of FI among student-athletes via a series of online focus groups. Unlike comparable studies investigating FI among collegiate student-athletes, this study collected insights from the perspectives of athletic professionals working with those athletes. Furthermore, this study provides a robust view and unique findings of the intertwining systems-level factors on the individual, team/Athletic Department, and university/societal tier.

### 4.1. Social Determinants of FI Among Collegiate Student-Athletes

Study participants self-reported being knowledgeable about the definitions of food security/insecurity and the association between FI and physical health, mental health, and athletic performance. Emerging social determinants of FI included financial challenges (personal, team/Athletic Department, and university), competing priorities, cultural/societal impacts, limited life skills, and the food environment.

Financial challenges precipitated as the primary contributing factor in six other studies that invested FI among collegiate student-athletes, combined *n* = 1132, 71.5% female, 65.5% non-Hispanic white [2,5,23,57,58,59]. Other studies concluded that financial strain was typical; student-athletes struggled with insufficient funds to meet basic needs [4,5,58,59,60,61]. Inadequacy of the student-athlete stipend to ensure adequate dietary intake has also been previously reported [2,5,23,57,58]. In the Brown et al. study, participants reported that the university meal plan was too expensive [23]. Whereas Vento et al. found that to be true regardless of sport or team, the findings of both Misener et al. and this study found that team budgetary inequities yielded differences in FI risk based on sport [59,61]. A student-athlete’s housing situation was also identified as a contributing factor in several studies [3,5,23,62]. In a survey conducted in the fall of 2019, almost 14% of Division I student-athletes reported experiencing homelessness in the past year [3]. Parallel with that finding, Douglas et al. and Anziano found that limited kitchen access and lack of cooking equipment were additional contributing factors [2,35]. In a review article (*n* = 47 studies), student-athletes living off-campus surfaced as a contributing factor to FI risk [63]. In the Lumina study, student-athletes who did not work during the season were at higher risk for FI [3]. Of note, NCAA rules prohibit employment for scholarship students during the season, and coach expectations are that non-scholarship counterparts will also not work [3].

Competing priorities was another powerful FI determinant for collegiate student-athletes (combined *n* = 1269, 67.4% female, 78.4% non-Hispanic white) reported by other researchers [2,5,23,35,57,60,63]. Specific subthemes included balancing commitments (athletic, academic, work, etc.) and scheduling conflicts (games and practice during dining room hours) [2,5,8,34,35,57,60,63]. Brauman et al. surveyed student-athletes (*n* = 169) from NCAA Division I public universities in the midwestern US regarding barriers to eating a healthy diet; a lack of time emerged as one of the top five reasons student-athletes struggle with FI [60]. The competing priorities’ theme also emerged regarding time and dining hall access limitations [8]. Reinforcing that circumstance, a study evaluating diet quality among NCAA Division I student-athletes (*n* = 94) found that only 2.1% of participant dietary intakes met the Dietary Guidelines for Americans [64].

Cultural/societal factors in this study included individual demographics intertwined with the team/Athletic Department and university/societal cultures (combined *n* = 1653, 44.2% female, 70.6% non-Hispanic white) [4,9,10,23,35,61,65,66]. Five studies reported that personal identity, student race or ethnicity, and gender were contributing factors; specifically, Black and Hispanic male student-athletes were at higher risk for FI [5,10,23,61,65]. Socioeconomics also emerged as a contributing factor; being food insecure before college, on financial aid, and a first-generation college student increased FI risk [4,61,66]. Chimera found that student-athletes at urban schools were at greater risk than those at rural schools; interestingly, this is contrary to findings regarding the general student population [5,9]. Unique findings in this study included individual food preferences and familiar items regardless of cost and financial security.

Eight other studies concluded that the food environment contributed to FI among student-athletes (combined *n* = 1588, 59.2% female, 76.7% non-Hispanic white) [5,8,23,35,58,60,61]. A noteworthy theme regarding food availability was the inadequacy of college meal plans to meet student-athlete energy (kcaloric) needs. Brown et al. and Misener et al. both found that athletes reported running out of meal swipes before the end of the month; Reader et al. reported that meal plans did not meet the elevated energy needs of athletes [5,23,61]. Easy access to unhealthy foods on campus and lack of access to healthy options in the dining room were examples of accessibility themes [35,60]. A food proximity issue, highlighted by Anziano, was the lack of transportation needed to purchase healthy options [35].

Limited life skills emerged as contributors to FI among student-athletes in two other studies (combined *n* = 247, 80.8% female, 83.2% non-Hispanic white) [2,60]. Knowledge and/or skill gaps in nutrition, shopping, and food preparation were identified as FI risk factors [2,60]. Unique to this study was the need for honing life skills in money and time management.

### 4.2. Systems-Level Determinants of FI Among Collegiate Student-Athletes

This study expands the understanding of the FI experience for student-athletes. Most of the findings from past studies identified individual student-athlete factors. Participants in this study pointed out that team/Athletic Departments and university/societal factors impact FI risk among student-athletes. System-level financial challenges emerged as a theme in this study. Anecdotal evidence reveals that Athletic Department budgets are complex and there are considerable variabilities across schools, especially regarding sources of funding for nutrition-related programs and services. Furthermore, budgetary models and allocations vary based on the NCAA Division [7].

Inadequate monetary amounts of stipends transcend the individual student as a FI contributing factor. For example, since stipends are based on meal-plan options, if the dining hall budget only affords inexpensive, low-quality options, stipends will likely be inadequate to purchase the quantity and quality food options required for optimal athletic performance. This reality emerged as a theme; indeed, some participants noted that student-athletes opt not to eat at the university. *“Another one is the quality of our dining hall…They always complain about the quality, and so they don’t choose to eat there when it is a resource on campus.”* (006) Furthermore, the practice of basing the amount of the stipends on the consumption of an average college student does not account for the excess kcalories required for student-athletes to meet practice and performance needs.

Another financial factor that emerged in this study was the potential positive benefit of the NIL policy. This source of individual student-athlete revenue, however, might have detrimental consequences for AthleticDepartments [67]. Dees et al. suggest that sponsors who historically supported an Athletic Department might shift funding to individual student-athletes, which could reduce the department budget [67]. The authors also note that student-athletes in revenue-generating male sports (football, basketball) will likely benefit more from NIL, and female student-athletes will probably reap fewer financial benefits [67]. Echoing this sentiment, Banks et al. investigated the NIL valuation among the top 100 student-athletes in the USA [68]. Top NIL earners were mostly male, black, football or basketball players (94%, 68%, 76%, and 22%, respectively [68]. Given studies have found that Black males are at greater risk for FI, this may help reduce prevalence among some high-risk student-athletes [10,23,35,61,65]. Thus, in the future, there might be a shift in the demographics of food insecure student-athletes.

Competing priorities on a systems level included Athletic Departments/universities opting to use budget funding for other priorities rather than hire a sports dietitian. Furthermore, in this study, participants were aware that practice times conflicted with dining hall hours; eating was, thus, a low priority for Athletic Departments and universities.

Cultural/societal factors also precipitated on a systems level. On the team/department and university tiers, stigma emerged as a theme. Unlike other food insecure populations, however, stigma relates to the assumption that student-athletes are given everything they need (scholarship funds/stipends, snacks, etc.); thus, they must not be food insecure. This cultural reality can make it difficult for student-athletes to admit they need help. Furthermore, the staff turnover can inhibit the ability of student-athletes to develop a trusting relationship with athletic professionals about their need for help. The finding that participants had ≥7 years of collegiate athletic department work experience, and most had been in their current position for <3 years reflects the turnover phenomenon occurring in university Athletic Departments. Work-life imbalances, long hours, juggling politics and bureaucratic policies, and low salaries, for example, have been demonstrated to exacerbate turnover among college athletic professionals [69,70]. Though staff turnover did not precipitate as a contributing factor, participant responses suggest it may play a role.

The food environment is highly controlled by most universities, and the limitations for student-athletes are significant. Universities with more economical dining hall plans are not offering student-athletes the quality, nutrient-dense options they require, which is akin to individuals buying inexpensive, convenience-type foods. Also, as noted above, basing the stipend on the average plan does not compensate for the excess calories required for optimal athletic performance. To address this issue, as well as conflicts with dining hall hours, some universities have dedicated athlete dining halls [71]. Furthermore, in these specialized dining facilities, sports dietitians help design personalized meal plans for student-athletes [71]. These nutrition experts, for example, can help international student-athletes address access challenges for culturally relevant foods.

In this study, the multi-factorial determinants of FI among student-athletes emerged, mitigation strategies that better address contributing factors unique to student-athletes are warranted. Based on the findings of this study, it is likely that team/Athletic Department and university budgetary decisions impact student-athlete utilization of FI mitigation programs. Resource inequities across teams on FI among student-athletes surfaced as an additional area for exploration. Future research is also needed on the impact of the 2024 NCAA Name, Image, Likeness (NIL) policy on FI status among student-athletes, as well as on athletic department budgets and non-sponsored food insecure student-athletes. Furthermore, the need for studies involving collegiate student-athletes with more diverse backgrounds emerged. At the time of publication of this study, studies in the literature reflected the experience of a homogeneous sample of student-athletes, primarily non-Hispanic white females.

### 4.3. Strengths and Limitations

The phenomenological design of this study provided rich data on the multi-factorial social determinants of FI among student-athletes. This methodology, however, coupled with the purposeful sample, can impact the transferability of the findings. One strength was the exploration of not just individual factors, but team/thletic epartment and university/societal-related contributing factors. The study employed coding and reporting best practices to help reduce the risk of bias via the researchers’ lens. The qualitative methodology, however, lacks the rigor of empirical, quantitative study designs. Study findings also reflect a period in which some schools had student-athletes on COVID-19-related Season of Competition and Extension of Eligibility waivers, which might have impacted the severity of resource limitations that emerged on individual, team/Athletic Department, and university/societal tiers.

## 5. Conclusions

The 2022–2027 Big Sky Conference Strategic Plan introduces new requirements ensuring optimal nutrition for student-athletes [72]. All schools must provide fueling stations offering snacks and nutrition education for student-athletes by the 2023–24 season. Minimum funding of $50,000 to sustain fueling stations is required by the 2024–2025 season, and all student-athletes must have access to a nutritionist by the 2024–25 season [72]. Given the interest in ensuring the nutritional needs of student-athletes are addressed, it was an advantageous time to investigate the social determinants of FI among this population.

This study demonstrated that determinants of FI among collegiate student-athletes include individual, team/Athletic Department, and university/societal factors. Athletic Department and university policies and budgetary decisions emerged as potential antagonists on food security among student-athletes. For student-athletes living on campus with scholarships, scheduling conflicts and inadequate access to nutrient-dense food options arose as the key contributing factor. In contrast, for those living off campus, the inadequacy of the stipend for food and housing surfaced as the key contributing factor. Ultimately, the phenomenon of FI among student-athletes involves intertwined system issues; financial challenges, competing priorities, and cultural factors were the strongest contributors. The potential benefit of the NCAA 2024 NIL policy for high-risk student-athletes also surfaced; however, these individual sponsorships might negatively impact the team/Athletic Department and university budgets. FI mitigation strategies for student-athletes must go beyond simply addressing individual factors. Obtainment of food security among collegiate student-athletes requires system changes at the team/Athletic Department and university tiers.

## Figures and Tables

**Table 1:** School profiles and participant demographics.

Category		No. (%)
SCHOOL PROFILES		
Funding Source	Public	12 (100.0)
	Private	0 (0.0)
Geographic Location ^1^	Urban (>60,000)	8 (66.7)
	Micro Urban (<60,000)	2 (16.7)
	Rural (<50,000)	2 (16.7)
Size of Student Body ^2^	Medium (<3000–9999)	2 (16.7)
	Large (≥10,000)	3 (25.0)
	X-Large (>15.000)	7 (58.3)
Athletic Department 2022 Revenue ^3^	10–19 M	6 (50.0)
	20–29 M	5 (41.7)
	30–39 M	1 (8.3)
PARTICIPANT DEMOGRAPHICS		
Birth Sex	Male	10 (37.0)
	Female	17 (63.0)
Former Collegiate Athlete	Yes	11 (40.7)
	No	16 (59.3)
Profession	Athletic Administrator	3 (11.1)
	Athletic Trainer	14 (51.9)
	Sports Performance Coach ^4^	4 (14.8)
	Sports Dietitian	6 (22.2)
Years in Profession	<1 year	0 (0.0)
	1–3 years	5 (18.5)
	4–6 years	7 (25.9)
	7–10 years	5 (18.5)
	>10 years	9 (33.3)
	No response	1 (3.7)
Years in Current Position	<1 year	4 (14.8)
	1–3 years	12 (44.4)
	4–6 years	3 (11.1)
	7–10 years	4 (14.8)
	>10 years	4 (14.8)

^1^ US Health Resources Service Administration; ^2^ Carnegie Classification; ^3^ Per school websites (see reference list); ^4^ Strength, conditioning, and nutrition performance coaches.

**Table 2:** Individual factors contributing to FI among student-athletes as perceived by collegiate athletic professionals.

Code	No. (%) *	Sample Quote
Subtheme #1: Financial		
Limited finances	17/27 (63.0)	“*I think there are people missing meals because they just don’t have money.*” (026)
Cost of living	6/27 (22.2)	“*When they run the numbers…they look at the dining halls, they base scholarship money on those numbers. It doesn’t necessarily translate to the expense of the community where they live…*” (023)
Affordability of healthy food	3/27 (11.1)	“*I would say those international brands are probably going to be pricier products, too. So, they’re using more of their budget.*” (009)
Resource distribution	3/27 (11.1)	“*…a handful of them don’t get stipends or other support.*” (012)
Subtheme #2: Limited Life Skills		
How to eat healthy	15/27 (55.6)	“*Another thing is lack of skills and knowledge about what is a healthy food.*” (014)
Shopping and meal prep	15/27 (55.6)	“*They’re very motivated to want to cook healthier and shop healthier. But skills and finances impact how much they can really do and how much time they have to even learn about those things.*” (020)
Money management	12/27 (44.4)	“*I think with skill-building opportunities, we should look at budgeting… one thing we could improve upon… is just talking about what… you should be putting your money towards, looking into how much those things actually cost, and what your budget would need to look like month to month in order to have a successful diet.”(022)*
Time management	10/27 (37.0)	“*While I do agree with the competing priorities side of it, with practices and juggling everything, that’s more time management.*” (026)
Subtheme #3: Competing Priorities		
Balancing commitments	7/27 (25.9)	“*... just not having enough time because of having to go to the weight room, practice, class tutoring, and all that stuff. They just didn’t have time to fit in time to eat.*” (019)
Eating is low priority	7/27 (25.9)	“*So, for them, it’s just balancing their schedule about what’s most important, I don’t have time to eat as much as I should or in that time frame.*” (006)
Convenience	4/27 (14.8)	“*What I see most frequently is the selection of convenience foods instead of nutrient-dense foods.*” (021)
Subtheme #4: Cultural (Food Preferences/Familiarity)		
Stigma (− sentiment)	12/27 (44.4%)	“*I’ve had multiple times, where athletes approach me and ask me about food and snacks. The athletes rely more on us, the front-line people, rather than administration or their coaches, because there’s just a stigma about it.*” (028)
Stigma(+ sentiment)	6/27 (22.2%)	“*I feel more and more that not just among the student-athletes, but even among the general population of students, there’s less stigma around the food security resources. I feel like students are way more open to it.*” (019)
Socio-economics	8/27 (29.6)	“*I have my group... it could be socioeconomic. Because the foods that they eat at home may not be the healthiest. And that’s just all they’re used to eating.*” (009)
Personal identity	7/27 (25.9)	“*I see American, European, and Asian students are way different with what they choose as a meal. And then also, balance and nutrition are way different…*” (014)

* Number (percent) of times code was mentioned by focus group participants.

**Table 3:** Teams/athletic department-related factors contributing to FI among student-athletes as perceived by collegiate athletic professionals.

Code	No. (%) *	Sample Quote
Subtheme #1: Financial		
Budget	10/27 (37.0)	“*I think finances are the biggest problem... as budget cuts come around … food is, unfortunately, the first thing to go as finances are limited.*” *(027)*
Insufficient stipend	9/27 (33.3)	“*The hardest thing for me is the difference between our headcount or full-ride athletes and those that are on equivalency, all the way to those that are walk-ons… I do think there’s a big difference for students who are getting a stipend every month to pay for their room and board, versus those who are on equivalency and maybe just having their books covered. There’s just a big disparity between the resources they have to buy food.*” *(023)*
Resource distribution	8/27 (29.6)	“*For us, it’s probably more team by team within athletics… what’s the budget for each team? What can they afford to do?*” *(007)*
Cost of food	3/27 (11.1)	“*…food is, unfortunately, the first thing to go as finances are limited*” (027)
Subtheme #2: Competing Priorities		
Eating is low priority	10/27 (37.0)	“*We’ve really, really, really tried to build up our fueling center… Some coaches have different philosophies about dipping into their sports accounts to help fund their fueling station…*” *(026).*
Inadequate fueling station funds	8/27 (22.2)	“*I left the [my previous] university… in December of last year, and…they just had a room that was open, and it was like, “This is your fueling station.” But really, it was snacks from Costco. It wasn’t even full of food…*” (010)
Nutrition programs, hiring a dietitian is low priority	5/27 (18.5)	“*We’re missing a Director of Sports Nutrition. It’s a huge piece that we’re missing, having somebody to direct this effort and all these inner workings and for someone to align it all. Hire a dietitian or two. That would be super cool.*” *(019)*
Subtheme #3: Cultural		
Dept. culture (+ sentiment)	6/17 (22.2)	“*…our student-athletes need more accessibility to food and we’re trying to push that... We’ve started stocking areas, especially high traffic areas like our weight room…we are looking for a fueling station…it has been a huge push for us… we’ve identified an area that our student-athletes need and we’re trying to move in the right direction*” *(008)*
Dept. culture (− sentiment)	5/27 (18.5)	“*The biggest frustration… as an athletic trainer, I did not have the administrative support from a lot of key individuals… I’d say with nutrition, they didn’t see the investment and the payoff for buying more food…*” *(026)*

* Number (percent) of times code was mentioned by focus group participants.

**Table 4:** University and societal determinants of FI among student-athletes as perceived by collegiate athletic professionals.

Code	No. (%) *	Sample Quote
Subtheme #1: Financial		
Quality of options	10/27 (37.0)	“*Another one is the quality of our dining hall…They always complain about the quality, and so they don’t choose to eat there when it is a resource on campus.*” (006)
Insufficient stipend	9/27 (33.3)	“*We probably have the most resources available to us with our football team, but we run into the same issues. The cost of living in our town has got ridiculous. I don’t think their stipend checks are enough to cover what they need…the majority is going to rent…they’re trying to stretch the limited funds they have left.*” *(024)*
Cost of living	4/27 (14.8)	“*The cost of living in our town has gotten ridiculous. I don’t think their stipend checks are enough to cover what they actually need.*” *(024)*
Subtheme#2: Competing Priorities (Food Environment Reflects Eating is Low Priority)		
Availability	7/27 (25.9)	“*…the second thing I noticed is practice times occur when our dining halls are open. We run into a lot of issues on the weekend… dining may not open until 11 or 12 o’clock. You know that day that getting breakfast is a big challenge.*” (002)
Access/proximity	4/27 (14.8)	“*…the international aspect is something that could be added…I feel like food insecurity impacts them in a different way. They have the finances, but they just feel like they don’t have access to the same things here as they do at home.*” (012)
Subtheme#3: Cultural/Societal		
Socio-economics	4/27 (14.8)	“*It’s a small community. It’s getting very expensive, and I think some of our other institutions are running into this, too. I don’t know how people live off campus and can afford rental and car insurance and food. I really don’t.*” *(026)*
Stigma	4/27 (14.8)	“*they’re afraid they’ll be stigmatized if they ask for that type of assistance*” (002)
School culture	3/27 (11.1)	“*Honestly… the athletic department is trying to solve this problem by themselves. The school should do more… if they want to provide better service for student-athletes and other students as well.*” (014)
Social media, advertising	2/27 (7.4)	“*I think spending a lot of money on supplements …they buy these supplements for $100 instead of making a grocery run…they think they are making a decision that is important for their performance, but had they just spent that money on food, they would have seen a better impact.*” (005)

* Number (percent) of times code was mentioned by focus group participants.

## Data Availability

The dataset presented in this article is not readily available because of ethical concerns related to the small sample size. Requests to access the datasets should be directed to jeniferreader@isu.edu.

## Supplementary Materials

The following supporting information can be downloaded at: https://www.mdpi.com/article/10.3390/nu17142254/s1, Table S1: Consolidated criteria for reporting qualitative studies (COREQ) 32-item checklist.

## Abbreviations

The following abbreviations are used in this manuscript:

FI	Food insecurity
GPA	Grade Point Average
NCAA	National Collegiate Athletic Association
NIL	Name, image, likeness
