# Supplementary material for: Systemic Factors Fuel Food Insecurity Among Collegiate Student-Athletes: Qualitative Findings from the Running on Empty Study
Source: Nutrients. 2025 Jul 8;17(14):2254. doi: 10.3390/nu17142254 (PMC12298077; doi:10.3390/nu17142254)
Supplement: Supplementary file 1 [file nutrients-17-02254-s001.zip › nutrients-3725511-supplementary.pdf]

Supplementary Table S1: Consolidated criteria for reporting qualitative studies (COREQ) 32-item checklist.

Item No	Guide Questions/Description	Page #
**DOMAIN 1: RESEARCH TEAM AND REFLEXIVITY**		
**Personal Characteristics**		
1. Interviewer/ facilitator	Which author/s conducted the interview or focus group?	5
2. Credentials	What were the researcher's credentials? E.g., PhD, MD	5
3. Occupation	What was their occupation at the time of the study?	5
4. Gender	Was the researcher male or female?	5
5. Experience and training	What experience or training did the researcher have?	5
**Relationship with participants**		
6. Relationship established	Was a relationship established prior to study commencement?	5
7. Participant knowledge of the interviewer	What did the participants know about the researcher? e.g. personal goals, reasons for doing the research?	5
8. Interviewer characteristics	What characteristics were reported about the interviewer/facilitator? e.g. Bias, assumptions, reasons and interests in the research topic	5
**DOMAIN 2: STUDY DESIGN**		
**Theoretical framework**		
9. Methodological orientation and Theory	What methodological orientation was stated to underpin the study? e.g. grounded theory, discourse analysis, ethnography, phenomenology, content analysis	4
**Participant selection**		
10. Sampling	How were participants selected? e.g., purposive, convenience, consecutive, snowball	4
11. Method of approach	How were participants approached? e.g., face-to-face, telephone, mail, email	4
12. Sample size	How many participants were in the study?	5
13. Non-participation setting	How many people refused to participate or dropped out? Reasons?	5
14. Setting of the data collection	Where was the data collected? e.g., home, clinic, workplace	4
15. Presence of nonparticipants	Was anyone else present besides the participants and researchers?	N/A
16. Description of sample	What are the important characteristics of the sample, e.g. demographic data, date, etc.?	4
**Data collection**		
17. Interview guide	Were questions, prompts, and guides provided by the authors? Was it pilot tested?	4
18. Repeat interviews	Were repeat interviews carried out? If yes, how many?	N/A
19. Audio/visual recording	Did the research use audio or visual recording to collect the data?	4
20. Field notes	Were field notes made during and/or after the interview or focus group?	N/A
21. Duration	What was the duration of the interviews or focus group?	4
22. Data saturation	Was data saturation discussed?	5
23. Transcripts returned	Were transcripts returned to participants for comment and/or correction?	5

Supplementary Table S1: Consolidated criteria for reporting qualitative studies (COREQ) 32-item checklist.

Item No	Guide Questions/Description	Page #
	**DOMAIN 3: ANALYSIS AND FINDINGS**	
	**Data analysis**	
24. Number of data coders	How many data coders coded the data?	5
25. Description of the coding tree	Did the authors provide a description of the coding tree?	N/A
26. Derivation of themes	Were themes identified in advance or derived from the data?	4-5
27. Software	What software, if applicable, was used to manage the data?	5
28. Participant checking	Did participants provide feedback on the findings?	N/A
	**Reporting**	
29. Quotations presented	Were participant quotations presented to illustrate the themes/findings? Was each quotation identified, e.g. participant number?	7-14
30. Data and findings consistent	Was there consistency between the data presented and the findings?	5-14
31. Clarity of major themes	Were major themes clearly presented in the findings?	5-14
32. Clarity of minor themes	Is there a description of diverse cases or a discussion of minor themes?	5-14

Source: Tong, A., Sainsbury, P., Craig, J. Consolidated criteria for reporting qualitative research (COREQ): a 32-item checklist for interviews and focus groups. Int J Qual Health C **2007,** 19, 349 – 357.
